# Supplementary material for: Mechanistic insights into histone recognition and H3K14 acetylation by the NuA3 histone acetyltransferase complex
Source: Nat Commun. 2025 Nov 29;17:342. doi: 10.1038/s41467-025-67049-0 (PMC12789522; doi:10.1038/s41467-025-67049-0)
Supplement: Supplementary file 1 — Supplementary Information [file 41467_2025_67049_MOESM1_ESM.pdf]

Supplementary Information

Mechanistic insights into histone recognition and H3K14 acetylation by the NuA3 histone acetyltransferase complex

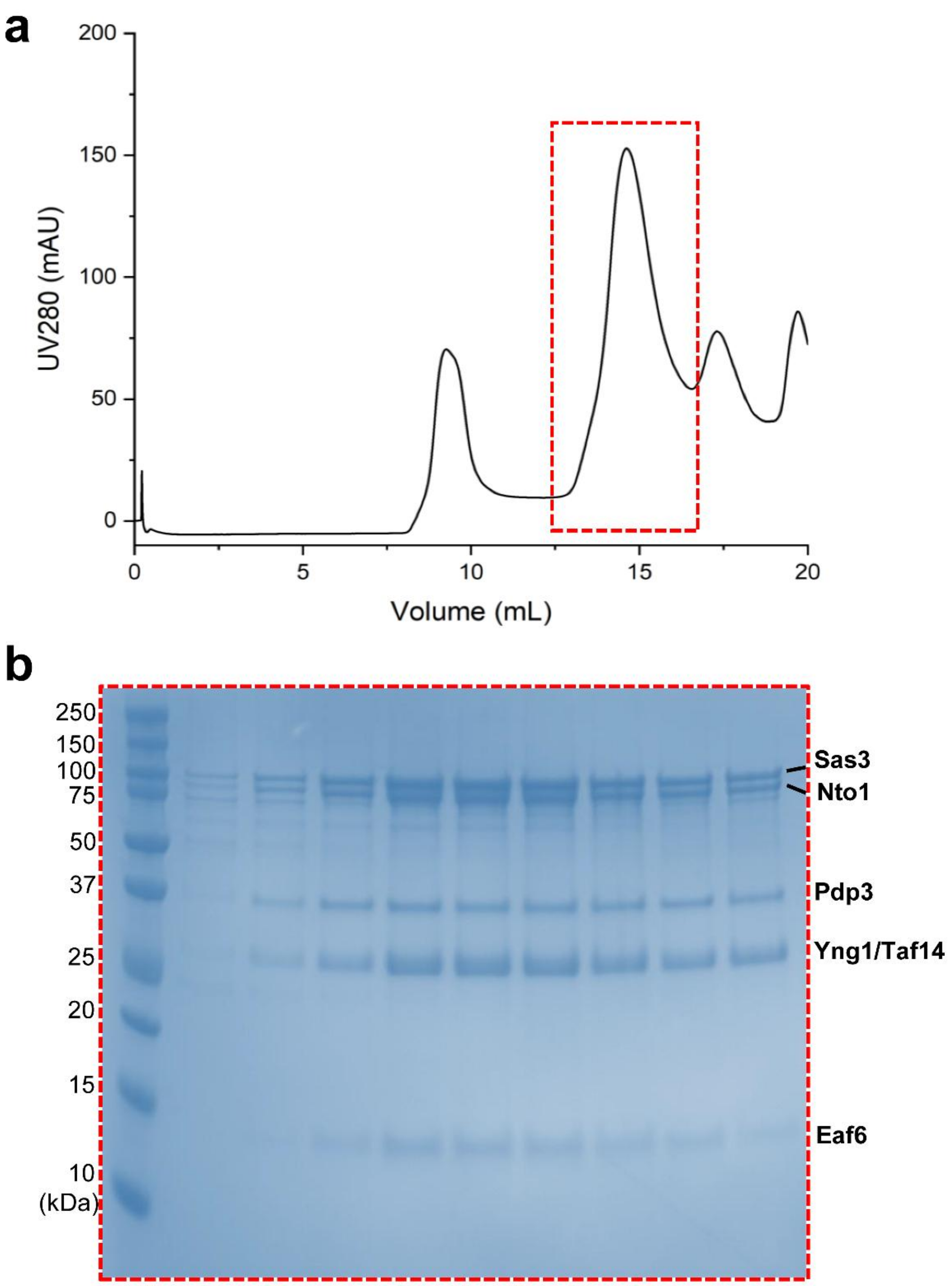

Supplementary Figure 1. Purification of the NuA3 complex. (**a**) Size-exclusion chromatography profile of the recombinant NuA3 complex. Source data are provided as a Source Data file. (**b**) SDS-PAGE analysis of the peak fractions collected from (a). Samples were resolved on a 12% polyacrylamide gel (Life Technologies) and stained with Coomassie blue. Individual subunits are labeled on the right.

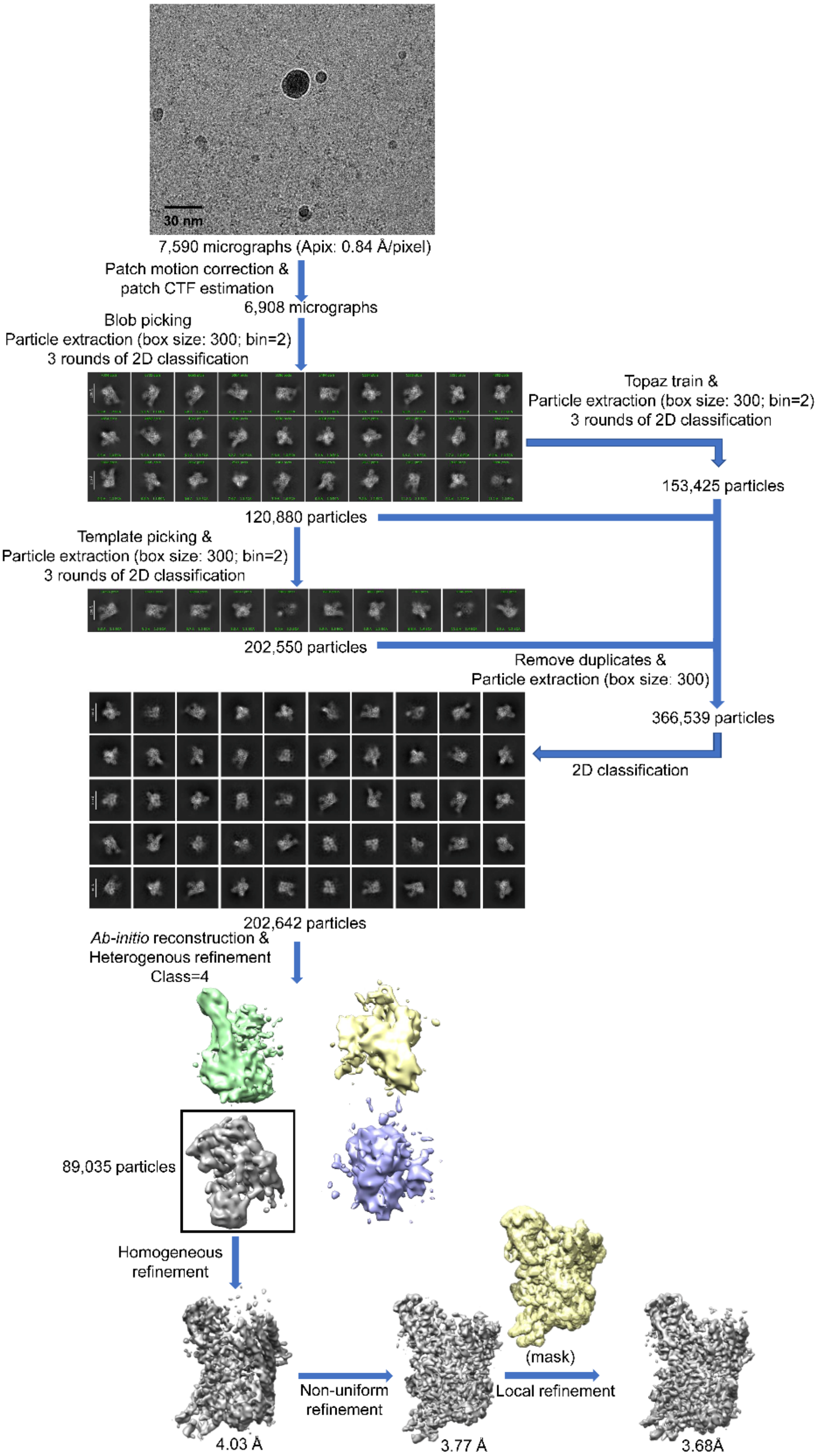

Supplementary Figure 2. Cryo-EM data processing workflow for the NuA3 complex in apo state. Data processing flowchart for the NuA3 complex in the apo state.

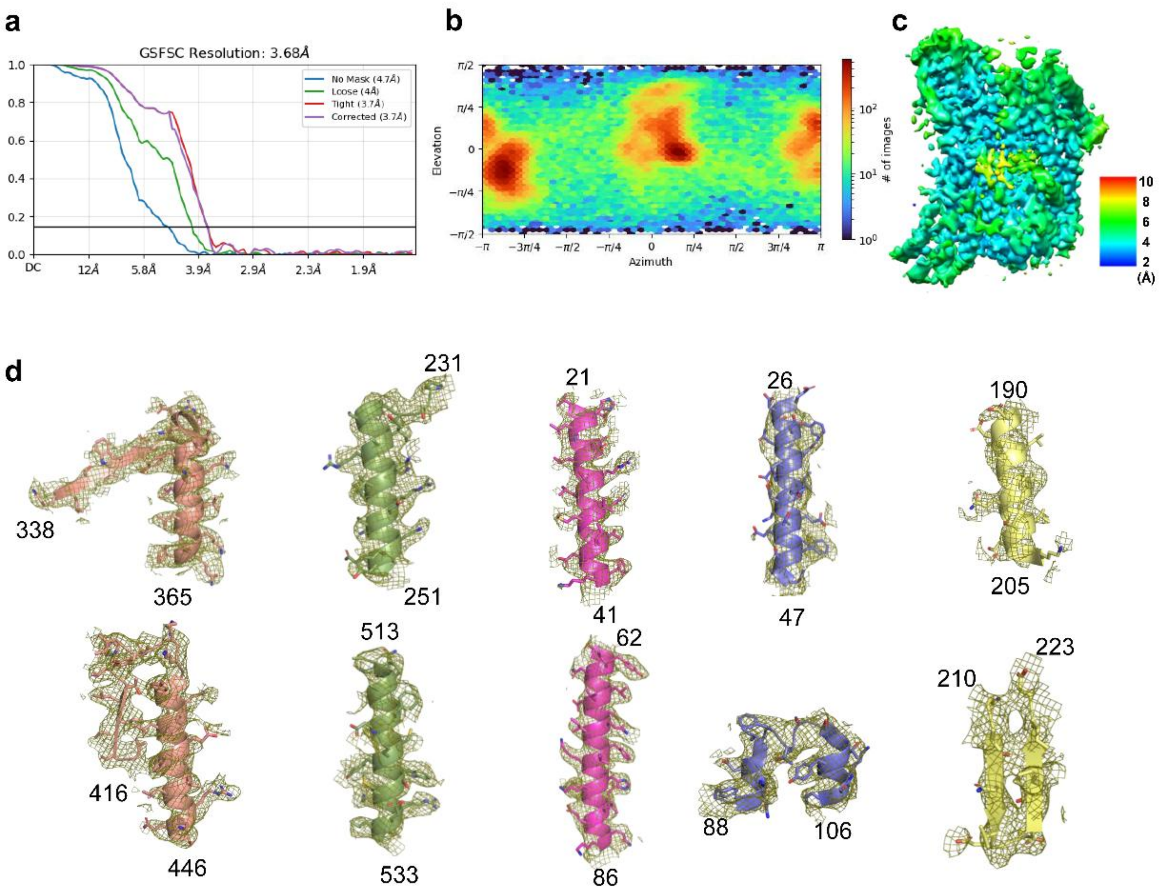

Supplementary Figure 3. Evaluation of the cryo-EM map of the NuA3 complex in apo state. (**a–c**) From left to right: the 0.143 gold-standard Fourier shell correlation (FSC) curve, angular distribution plot of particle orientations, and color-coded local resolution map of the apo NuA3 complex. (**d**) Representative regions of the cryo-EM density map.

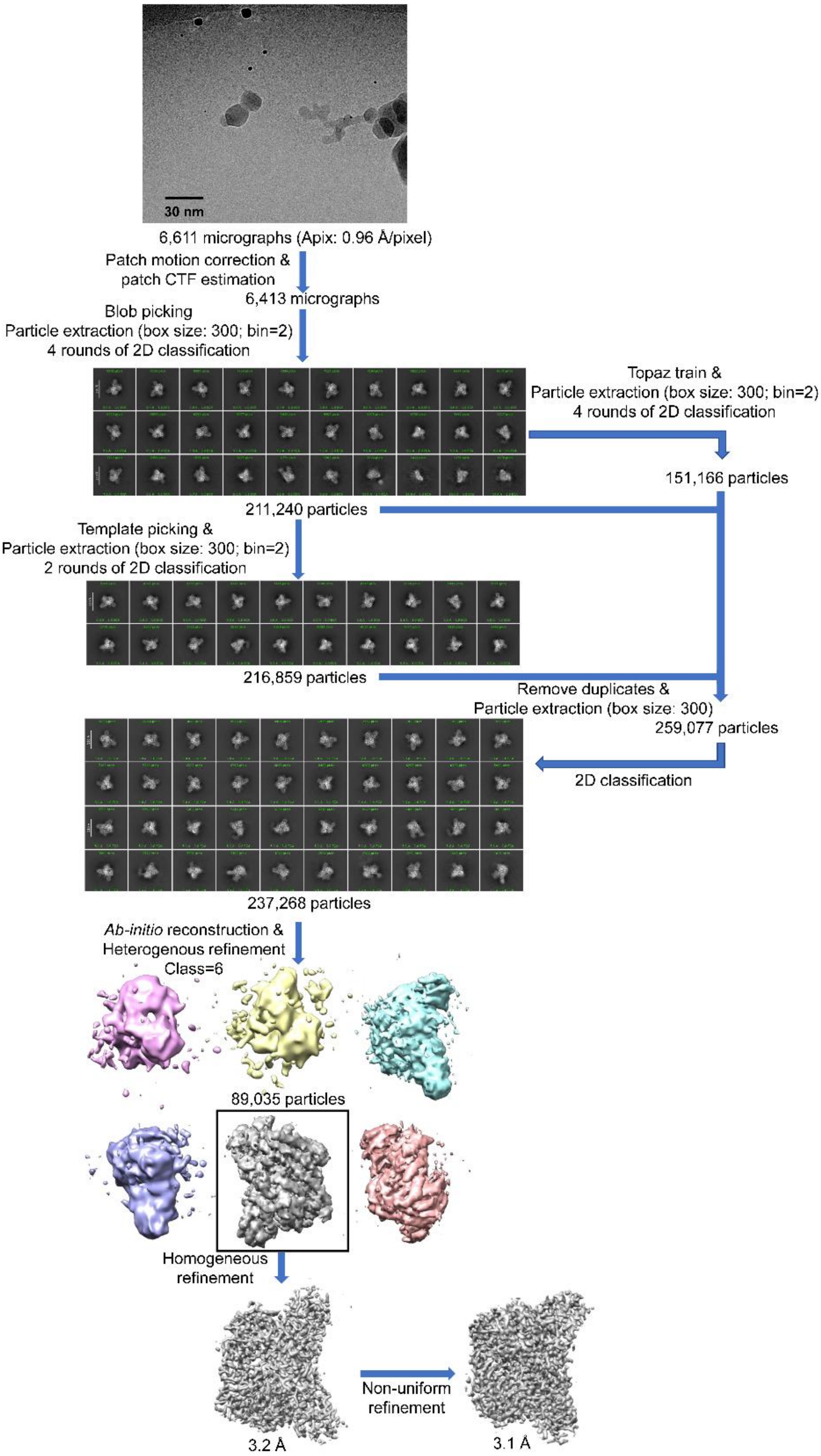

Supplementary Figure 4. Cryo-EM data processing workflow for the NuA3 complex bound to acetyl-CoA. Data processing flowchart for the NuA3 complex bound to acetyl-CoA.

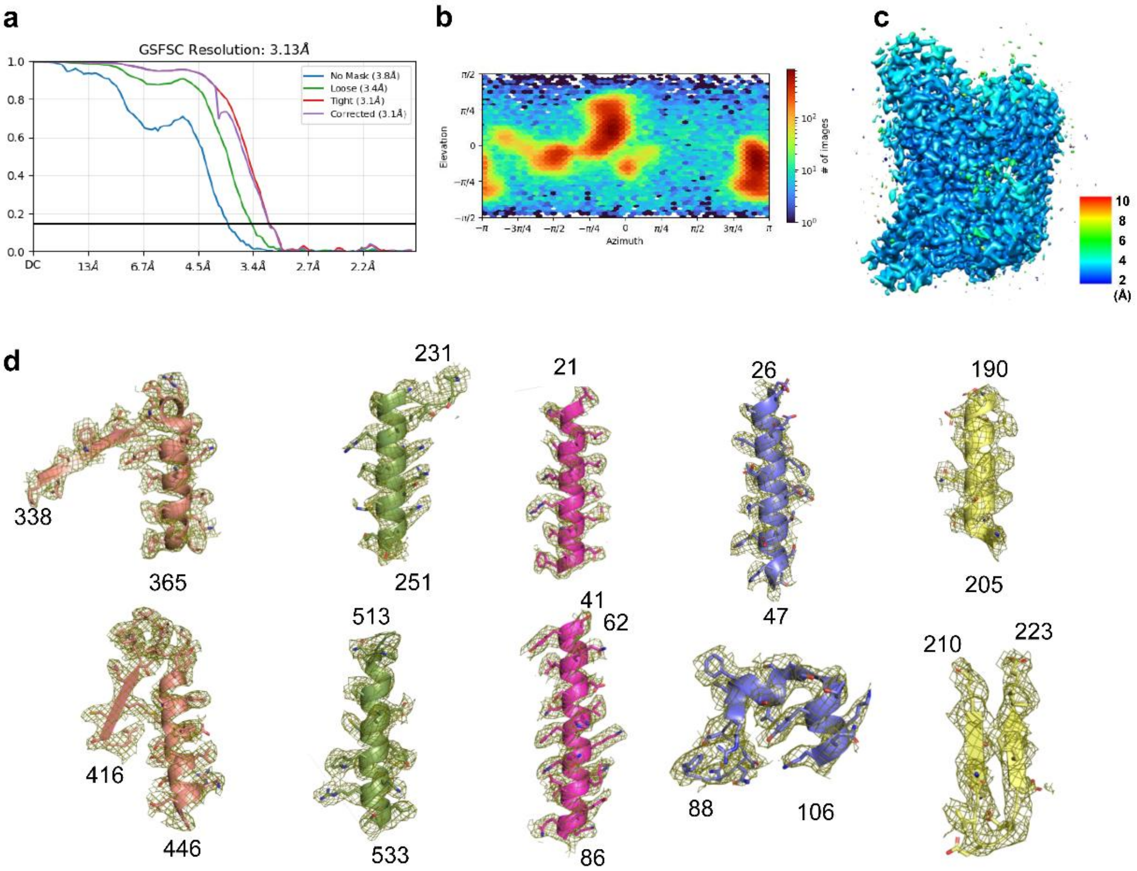

Supplementary Figure 5. Evaluation of the cryo-EM map of the NuA3 complex bound to acetyl-CoA. (**a–c**) From left to right: the 0.143 gold-standard Fourier shell correlation (FSC) curve, angular distribution plot of particle orientations, and color-coded local resolution map of the NuA3 complex bound to acetyl-CoA. (**d**) Representative regions of the cryo-EM density map.

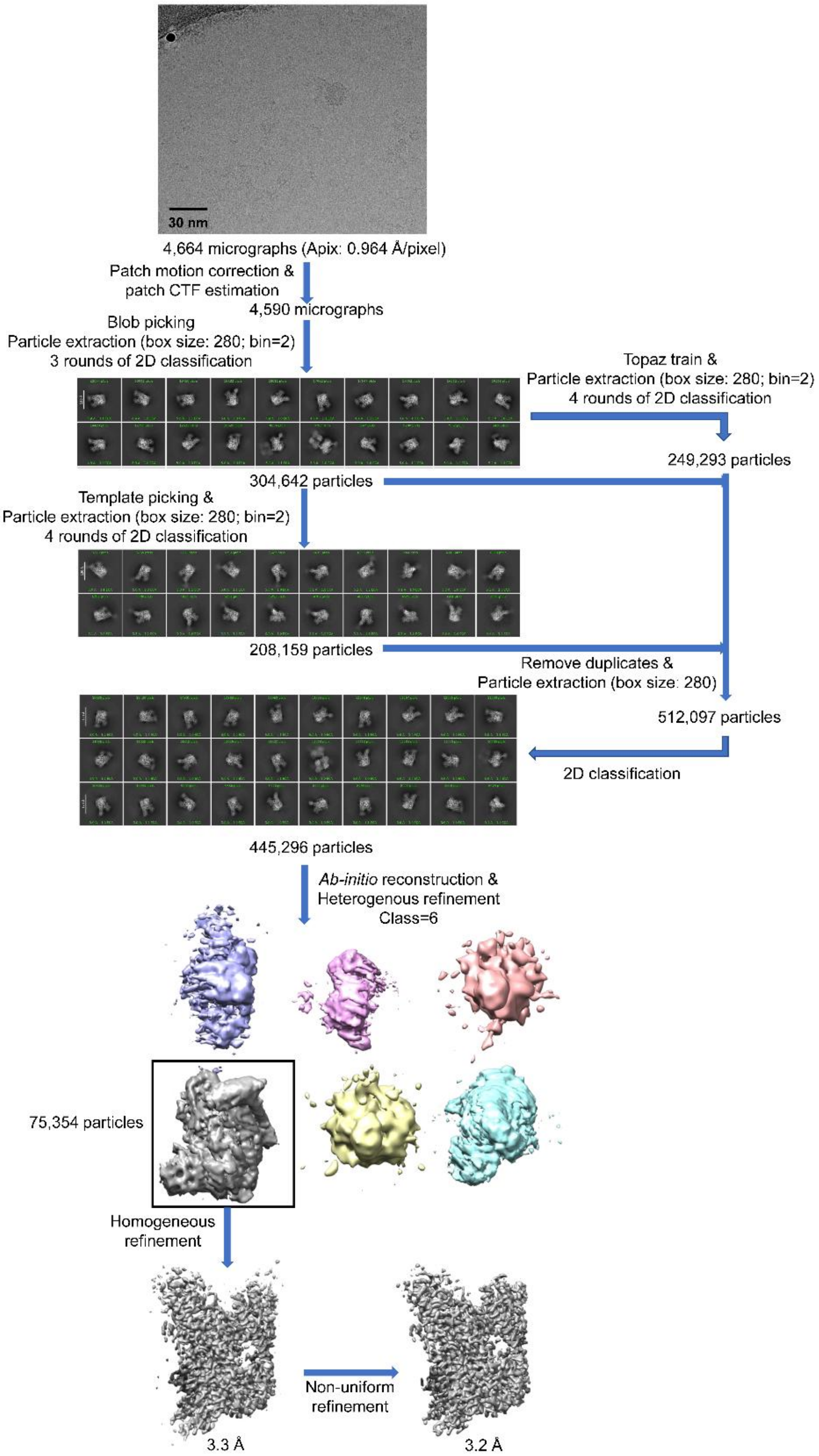

Supplementary Figure 6. Cryo-EM data processing workflow for the NuA3 complex abound to substrate and cofactor. Data processing flowchart for the NuA3 complex bound to the histone H3 tail and acetyl-CoA.

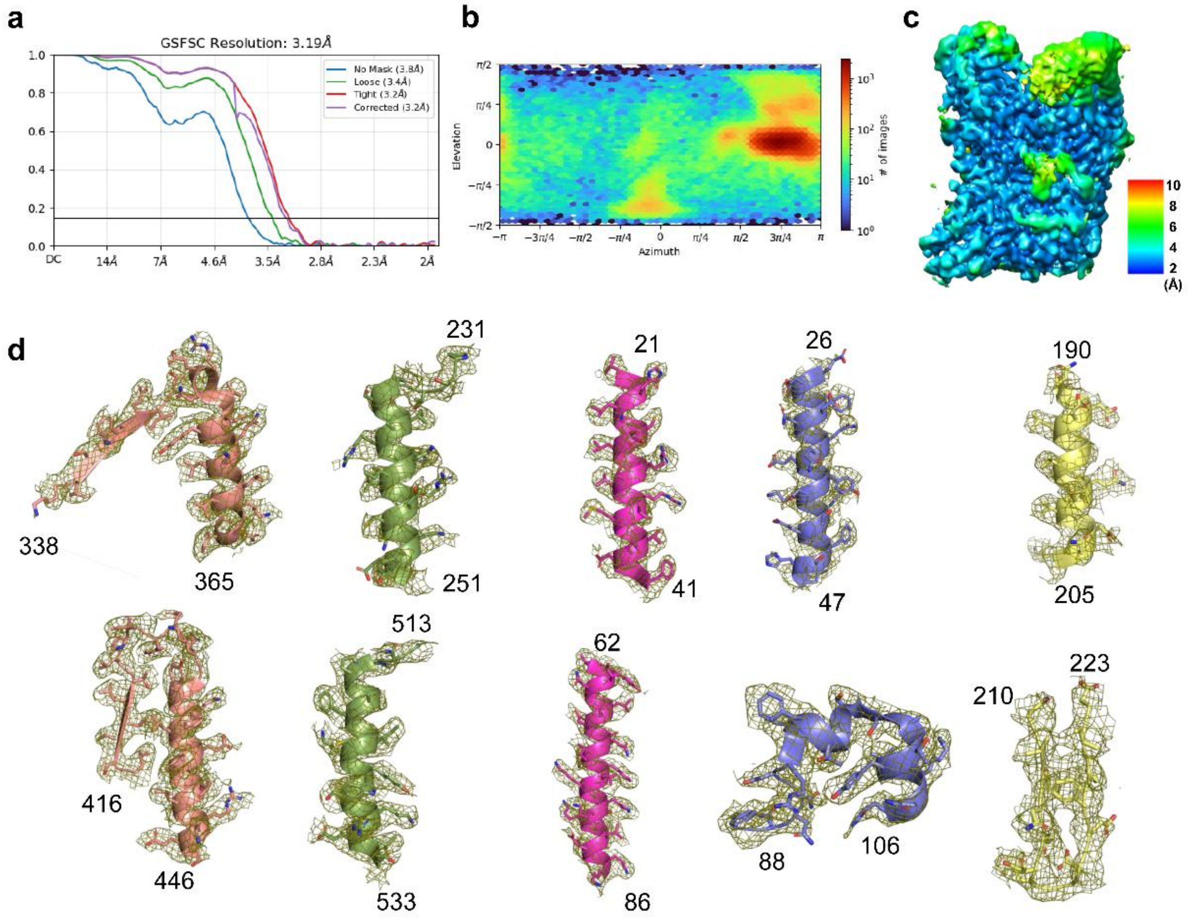

Supplementary Figure 7. Evaluation of the cryo-EM map of the NuA3 complex bound to histone tail and acetyl-CoA. (**a–c**) From left to right: the 0.143 gold-standard FSC curve, angular distribution plot, and color-coded local resolution map of the NuA3 complex in the histone tail/acetyl-CoA-bound state. (**d**) Representative regions of the cryo-EM density map.

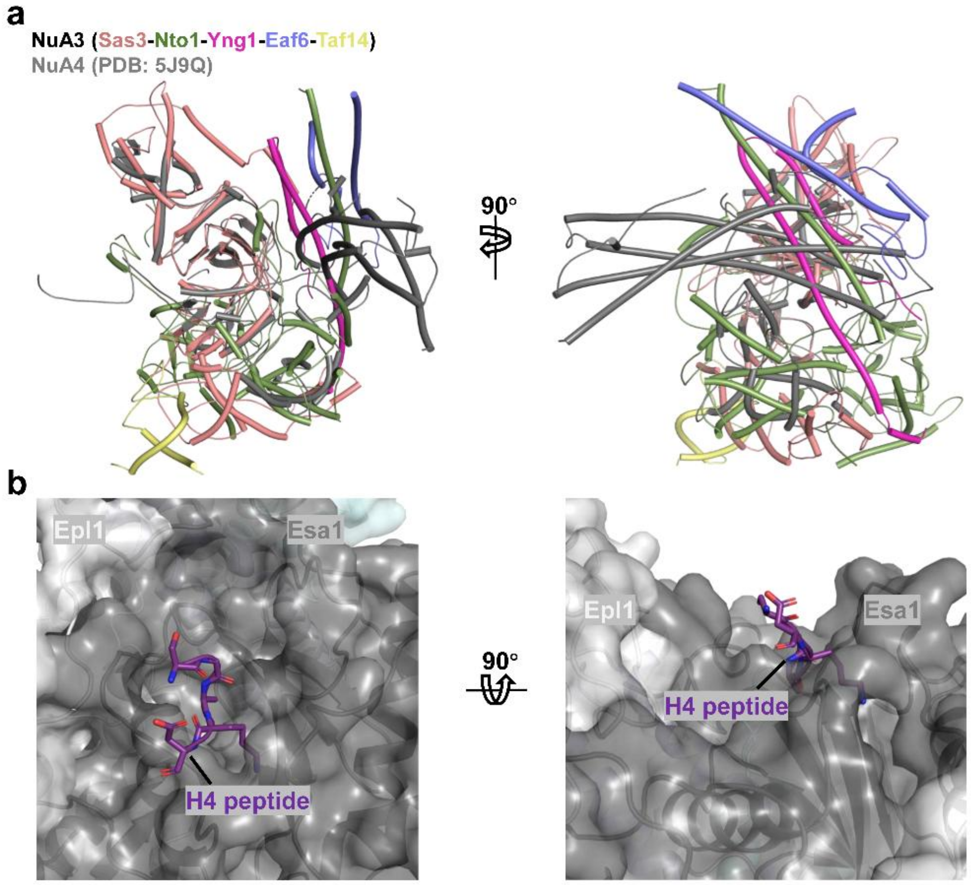

Supplementary Figure 8. Structural comparison between the NuA3 and NuA4 complexes. (**a**) Structural superposition of NuA4 onto NuA3. NuA4 is shown in gray, and NuA3 is color-coded as in Figure 1, rotated by 90°. (**b**) Histone tail binding cleft of the NuA4 complex. Epl1 and Esa1 are shown as surface, and the Htz1 histone peptide is shown in ribbon representation, rotated by 90°.

Supplementary Table 1. Cryo-EM data collection, refinement, and validation statistics.

Protein complex	Apo NuA3 complex	NuA3-acetyl-CoA	NuA3-H3-acetyl-CoA
EMDB	EMD-64513	EMD-65145	EMD-64517
PDB	9UUO	9VKW	9UUS
**Data collection and processing**			
Magnification	105,000	130,000	130,000
Voltage (kV)	300	300	300
Electron exposure ($e^-/Å^2$)	50	50	50
Defocus range (-μm)	1.3-2.5	1.3-2.5	1.3-2.5
Pixel size (Å)	0.84	0.96	0.96
Symmetry imposed	C1	C1	C1
Initial particle images (no.)	222,642	227,268	445,296
Final particle images (no.)	89,035	89,035	176,266
Map resolution (Å)	3.7	3.1	3.2
FSC threshold	0.143	0.143	0.143
Map resolution range (Å)	3.0-4.5	2.5-3.5	2.5-3.6
**Refinement**			
Initial model used	AlphaFold2-predicted	Apo NuA3	Apo NuA3
Model resolution (Å)	3.7	3.1	3.2
FSC threshold	0.143	0.143	0.143
Model resolution range (Å)	3.0-4.5	2.5-3.5	2.5-3.6
Map sharpening B factor ($Å^2$)	145.6	132.0	113.6
Model composition			
Non-hydrogen atoms	11,029	10,739	10,798
Protein residues	1,343	1,301	1,311
Ligands	6	6	6
B factor ($Å^2$)			
Protein	113.3	139.5	107.6
Ligand	125.2	119.3	74.6
R.m.s. deviations			
Bond lengths (Å)	0.005	0.004	0.005
Bond angles (°)	0.681	0.579	0.680
Validation			
MolProbity score	2.29	2.70	2.64
Clashscore	20.9	9.9	10.7
Poor rotamers (%)	0	0	0
Ramachandran plot			
Favored (%)	92.3	91.9	93.2
Allowed (%)	7.7	8.1	6.8
Disallowed (%)	0	0	0

Supplementary Table 2. DNA primers used for construct design and mutagenesis.

Name	Sequence (5' to 3')
*SAS3*_F	ATGTCATTAACAGCAAACGACG
*SAS3*_R	TTATTCTTCGTCATCCTCTATTAGAGTTATTTTTC
*YNG1*_F	ATGGAACATCTCGCCAACG
*YNG1*_R	TTACTTTCTTCTTTTTTGCCGTTTTG
*NTO1*_F	ATGAATAGGGGATCATTAGATGATGG
*NTO1*_R	TTATCCGCTGTTAAGGATTAGCTTC
*EAF6*_F	ATGACTGATGAGCTGAAAAGTTATG
*EAF6*_R	TTAGTCGTTTTGTGATTGGCC
*TAF14*_F	ATGGTAGCTACAGTAAAAAGAACCATC
*TAF14*_R	TACTCGGTATTTTTCTTAACGTAGTCC
*PDP3*_F	ATGACAAAAGATATTAGAACAGGCG
*PDP3*_R	TCAAGGCGTAGGTAGCG
SAS3-Trunc._HR_F	AAGACTTGATCACCCGGGATCTCGAGATGGAACCTAATATTGAAGTCAGC G
*SAS3*_HR_R	GTTAGCCTCCCCCATCTCCCGGTACCTTACTTATCGTCGTCATCCTTGTAA TCACCCTGAAAATACAAATTCTCTTCTTCGTCATCCTCTATTAGAGTTATT
*NTO1*_HR_F	AAGACTTGATCACCCGGGATCTCGAGATGAATAGGGGATCATTAGATGAT GG
*NTO1*_HR_R	GTTAGCCTCCCCCATCTCCCGGTACCTTATCCGCTGTTAAGGATTAGCTT C
*TAF14*_HR_F	AAGACTTGATCACCCGGGATCTCGAGATGGTAGCTACAGTAAAAAGAACC ATCC
*TAF14*_HR_R	GTTAGCCTCCCCCATCTCCCGGTACCTTACTCGGTATTTTTCTTAACGTAG TCC
*EAF6*_HR_F	CGTCGACGAGCTCACTAGTCGCGGCCGCATGACTGATGAGCTGAAAAGT TATGAA
*EAF6*_HR_R	AGCTTGTCGAGACTGCAGGCTCTAGATTAGTCGTTTTGTGATTGGCCA
*YNG1*_HR_F	CGTCGACGAGCTCACTAGTCGCGGCCGCATGGAACATCTCGCCAACGA
*YNG1*_HR_R	AGCTTGTCGAGACTGCAGGCTCTAGATTAGTGGTGATGGTGATGATGGG GCCCCTGGAACAGAACTTCCAGCTTTCTTCTTTTTTGCCGTTTT
*PDP3*_HR_F	CGTCGACGAGCTCACTAGTCGCGGCCGCATGACAAAAGATATTAGAACAG GCGA

*PDP3*_HR_R	AGCTTGTCGAGACTGCAGGCTCTAGATCAAGGCGTAGGTAGCGCTT
SAS3(L369R/WT)_F	AACCAAAATAAAATGGTTTTTATATGTGAGTTC
SAS3(L369R/WT)_R	TATTTTAAGCTAAGAGACGAATTTAGTTTG
SAS3(E452Q)_F	ACGATGTTGAACCGTTTATATTCTATATTC
SAS3(E452Q)_R	TATTTTAAGCTAAGAGACGAATTTAGTTTG

Supplementary Table 3. Antibodies used in this study.

Antibody	Clone	Source	Catalog number	Dilution used
Anti-H3K14ac monoclonal antibody	D4B9	Rabbit	Cell signaling #7627T	1:1000
Anti-H3K14ac monoclonal antibody	EP964Y	Rabbit	Abcam #ab52946	1:5000
Anti-histone H3 monoclonal antibody	G22B21	Rabbit	Selleck #F0057	1:2000
Anti-rabbit IgG, HRP-linked antibody	-	Goat	Cell signaling #7074S	1:3000
Anti-rabbit IgG H&L (HRP) antibody	-	Goat	Abcam #ab6721	1:5000
