# Supplementary material for: Mechanistic insights into histone recognition and H3K14 acetylation by the NuA3 histone acetyltransferase complex
Source: Nat Commun. 2025 Nov 29;17:342. doi: 10.1038/s41467-025-67049-0 (PMC12789522; doi:10.1038/s41467-025-67049-0)
Supplement: Supplementary file 2 — Reporting Summary [file 41467_2025_67049_MOESM2_ESM.pdf]

nature portfolio

Corresponding author(s): Roger D. Kornberg, Heqiao Zhang

Last updated by author(s): Nov 7, 2025

Reporting Summary

Nature Portfolio wishes to improve the reproducibility of the work that we publish. This form provides structure for consistency and transparency in reporting. For further information on Nature Portfolio policies, see our Editorial Policies and the Editorial Policy Checklist.

Statistics

For all statistical analyses, confirm that the following items are present in the figure legend, table legend, main text, or Methods section.

n/a	Confirmed	
☐	☒	The exact sample size (n) for each experimental group/condition, given as a discrete number and unit of measurement
☐	☒	A statement on whether measurements were taken from distinct samples or whether the same sample was measured repeatedly
☒	☐	The statistical test(s) used AND whether they are one- or two-sided<br>*Only common tests should be described solely by name; describe more complex techniques in the Methods section.*
☒	☐	A description of all covariates tested
☒	☐	A description of any assumptions or corrections, such as tests of normality and adjustment for multiple comparisons
☐	☒	A full description of the statistical parameters including central tendency (e.g. means) or other basic estimates (e.g. regression coefficient) AND variation (e.g. standard deviation) or associated estimates of uncertainty (e.g. confidence intervals)
☒	☐	For null hypothesis testing, the test statistic (e.g. F, t, r) with confidence intervals, effect sizes, degrees of freedom and P value noted<br>*Give P values as exact values whenever suitable.*
☒	☐	For Bayesian analysis, information on the choice of priors and Markov chain Monte Carlo settings
☒	☐	For hierarchical and complex designs, identification of the appropriate level for tests and full reporting of outcomes
☒	☐	Estimates of effect sizes (e.g. Cohen's d, Pearson's r), indicating how they were calculated

Our web collection on statistics for biologists contains articles on many of the points above.

Software and code

Policy information about availability of computer code

Data collection	EPU2.12, SerialEM
Data analysis	MotionCor2 1.2.1, CTFFIND v4.1.10, CryoSPARC 4.5.3, Phenix 1.17.1, COOT 0.9.8, PyMOL 2.1, UCSF Chimera 1.14, GraphPad prism 9.0, OriginPro 2025

For manuscripts utilizing custom algorithms or software that are central to the research but not yet described in published literature, software must be made available to editors and reviewers. We strongly encourage code deposition in a community repository (e.g. GitHub). See the Nature Portfolio guidelines for submitting code & software for further information.

Data

Policy information about availability of data

All manuscripts must include a data availability statement. This statement should provide the following information, where applicable:

- Accession codes, unique identifiers, or web links for publicly available datasets
- A description of any restrictions on data availability
- For clinical datasets or third party data, please ensure that the statement adheres to our policy

The cryo-EM reconstruction maps for the NuA3 complex in its apo form, bound to acetyl-CoA, and in a complex with both the histone H3 and acetyl-CoA have been deposited in the Electron Microscopy Data Bank (EMDB) under the accession codes EMD-64513 [https://www.ebi.ac.uk/pdbe/entry/emdb/EMD-64513], EMD-65145 [https://www.ebi.ac.uk/pdbe/entry/emdb/EMD-64515], and EMD-64517 [https://www.ebi.ac.uk/pdbe/entry/emdb/EMD-64517], respectively. Their respective coordinate files have been deposited in the Protein Data Bank (PDB) under the accession numbers 9UUO [http://doi.org/10.2210/pdb9UUO/pdb], 9VKM

[http://doi.org/10.2210/pdb9VKM/pdb], and 9UUS [http://doi.org/10.2210/pdb9UUS/pdb], respectively. All data are available in the manuscript or the supplementary materials. Any additional information required to reanalyze the data reported in this paper is available from the lead contact upon request. Source data are provided with this paper.

Research involving human participants, their data, or biological material

Policy information about studies with human participants or human data. See also policy information about sex, gender (identity/presentation), and sexual orientation and race, ethnicity and racism.

Reporting on sex and gender	No human research participants or human data were involved in this study.
Reporting on race, ethnicity, or other socially relevant groupings	No human research participants or human data were involved in this study.
Population characteristics	No human research participants or human data were involved in this study.
Recruitment	No human research participants or human data were involved in this study.
Ethics oversight	No human research participants or human data were involved in this study.

Note that full information on the approval of the study protocol must also be provided in the manuscript.

Field-specific reporting

Please select the one below that is the best fit for your research. If you are not sure, read the appropriate sections before making your selection.

☒ Life sciences ☐ Behavioural & social sciences ☐ Ecological, evolutionary & environmental sciences

For a reference copy of the document with all sections, see nature.com/documents/nr-reporting-summary-flat.pdf

Life sciences study design

All studies must disclose on these points even when the disclosure is negative.

Sample size	No statistical methods were used to predetermine sample size. Sufficient cryo-EM micrographs were collected on Titan Krios. Data processings in cryoSPARC yielded reconstruction maps at 3.0-3.7 angstrom, respectively, which are sufficient for interpretation of the experimental data and to build an atomic model.
Data exclusions	Particle sorting and exclusion during cryo-EM data processing in cryoSPARC followed standard procedures and the remaining particle numbers of all rounds are mentioned in the Methods section.
Replication	At least three replicates for protein purification. At least three replicates for cryo-EM analyses (different grid preparation). At least three replicates for enzymatic assays. All attempts at replication were successful.
Randomization	The cryo-EM data was analyzed according to the Gold-standard FSC.
Blinding	Investigators were not blinded to group allocation. It is not applicable to our study.

Reporting for specific materials, systems and methods

We require information from authors about some types of materials, experimental systems and methods used in many studies. Here, indicate whether each material, system or method listed is relevant to your study. If you are not sure if a list item applies to your research, read the appropriate section before selecting a response.

Materials & experimental systems

n/a	Involved in the study	
☐	☒	Antibodies
☐	☒	Eukaryotic cell lines
☒	☐	Palaeontology and archaeology
☒	☐	Animals and other organisms
☒	☐	Clinical data
☒	☐	Dual use research of concern
☒	☐	Plants

Methods

n/a	Involved in the study	
☒	☐	ChIP-seq
☒	☐	Flow cytometry
☒	☐	MRI-based neuroimaging

Antibodies

Antibodies used	Anti-FLAG G1 affinity resin was purchased from GenScript (Cat number: L00432-10; lot number: 2403K028); anti-H3K14ac (1:1000 dilution, Cell Signaling Technology, Cat# 7627T, Clone D4B9); anti-H3K14ac (1:5000, CST, Cat# ab52946, Clone 8EP964Y); Anti-histone H3 (1:2000, Selleck, Cat#F0057 , Clone G22B21); anti-rabbit IgG, HRP-linked antibody (1:3000, Cell Signaling Technology, Cat# 7074S); anti-rabbit IgG H&L (HRP) antibody(1:5000, Abcam Cat# ab6721).
Validation	Full validation reports for all primary antibodies listed above are available on the supplier websites.

Eukaryotic cell lines

Policy information about cell lines and Sex and Gender in Research

Cell line source(s)	High Five cells (BTI-TN-5B1-4) were purchased from Thermo Fisher Scientific (Invitrogen™, Cat. No. B85502).
Authentication	Authentication for the High Five insect cell line was performed by the supplier (Thermo Fisher Scientific) based on morphology and viability.
Mycoplasma contamination	The High Five cell line was tested negative for mycoplasma contamination by the supplier prior to shipment.
Commonly misidentified lines (See ICLAC register)	No commonly misidentified cell lines (as listed by ICLAC) were used in this study.

Plants

Seed stocks	No plants were used in this study.
Novel plant genotypes	No plants were used in this study.
Authentication	No plants were used in this study.
